# A transcriptome-wide association study of Alzheimer’s disease using prediction models of relevant tissues identifies novel candidate susceptibility genes

**DOI:** 10.1186/s13073-021-00959-y

**Published:** 2021-09-01

**Authors:** Yanfa Sun, Jingjing Zhu, Dan Zhou, Saranya Canchi, Chong Wu, Nancy J. Cox, Robert A. Rissman, Eric R. Gamazon, Lang Wu

**Affiliations:** 1 College of Life Science, Longyan University, Longyan, Fujian 364012 People’s Republic of China; 2 Cancer Epidemiology Division, Population Sciences in the Pacific Program, University of Hawaii Cancer Center, University of Hawaii at Manoa, Honolulu, HI 96813 USA; 3 Fujian Provincial Key Laboratory for the Prevention and Control of Animal Infectious Diseases and Biotechnology, Longyan, Fujian 364012 People’s Republic of China; 4 Fujian Provincial Universities Key Laboratory of Preventive Veterinary Medicine and Biotechnology (Longyan University), Longyan, Fujian 364012 People’s Republic of China; 5 Vanderbilt Genetics Institute and Division of Genetic Medicine, Department of Medicine, Vanderbilt University Medical Center, Nashville, TN 37232 USA; 6 Department of Neurosciences, University of California, San Diego, La Jolla, CA 92093 USA; 7 Veterans Affairs San Diego Healthcare System, San Diego, CA 92093 USA; 8 Department of Statistics, Florida State University, Tallahassee, FL 32306 USA; 9 Clare Hall, University of Cambridge, Cambridge, CB3 9AL UK; 10 MRC Epidemiology Unit, School of Clinical Medicine, University of Cambridge, Cambridge, CB2 0SL UK

**Keywords:** Alzheimer’s disease, Transcriptome-wide association study, Susceptibility genes, Etiology

## Abstract

**Background:**

Genome-wide association studies (GWAS) have identified over 56 susceptibility loci associated with Alzheimer’s disease (AD), but the genes responsible for these associations remain largely unknown.

**Methods:**

We performed a large transcriptome-wide association study (TWAS) leveraging modified UTMOST (Unified Test for MOlecular SignaTures) prediction models of ten brain tissues that are potentially related to AD to discover novel AD genetic loci and putative target genes in 71,880 (proxy) cases and 383,378 (proxy) controls of European ancestry.

**Results:**

We identified 53 genes with predicted expression associations with AD risk at Bonferroni correction threshold (*P* value < 3.38 × 10^−6^). Based on fine-mapping analyses, 21 genes at nine loci showed strong support for being causal.

**Conclusions:**

Our study provides new insights into the etiology and underlying genetic architecture of AD.

**Supplementary Information:**

The online version contains supplementary material available at 10.1186/s13073-021-00959-y.

## Background

Alzheimer’s disease (AD) is a common neurodegenerative disorder in the aging population [1]. The primary pathological feature of AD is characterized by aggregation of amyloid β peptides into extracellular plaques, as well as hyperphosphorylated tau into intracellular neurofibrillary tangles, accompanied by neuroinflammation, gliosis, and neurodegeneration [2]. The life quality of AD patients is significantly decreased because of severe impairment in individual executive and cognitive functions [3], which brings a substantial burden on not only the patients, but also their families, society, and the healthcare system [4]. It is estimated that in 2019, 5.8 million people that aged beyond 65 were diagnosed with AD in the USA, which had yielded a total expenditure of approximately 290 billion dollars for health care, long-term care, and hospice services [5]. To reduce the burden of AD, a better characterization of the etiology of AD is critically needed. Mutations in specific genes such as *APP*, *PSEN1*, *PSEN2*, *APOE*, and *TREM2* are reported to increase the risk of developing AD [6]. In addition, Genome-wide Association Studies (GWAS) have identified more than 56 common genetic loci associated with AD risk [7]. However, these loci can explain only a small fraction of the heritability of AD [8, 9]. Apart from conventional GWAS focusing on individual variants, there has been recent interest in transcriptome-wide association studies (TWAS) focusing on genetically predicted gene expression to gain additional insights into the genetic basis of complex traits and diseases [10]. This methodology integrates gene expression genetic prediction models built in reference datasets and large-scale disease GWAS datasets to identify novel candidate susceptibility genes whose genetically predicted expression levels are associated with the traits [11].

Several TWAS have already been conducted to identify candidate susceptibility genes for AD risk. In an earlier TWAS by Hao et al. involving 17,008 AD cases and 37,154 controls, 25 AD risk-associated genes were identified by leveraging gene expression prediction models of brain dorsolateral prefrontal cortex, adipose, and blood tissues [12]. Raj et al. leveraged dorsolateral prefrontal cortex (DLPFC) tissue gene expression prediction models and identified eight associated genes at novel loci by studying 25,580 cases and 48,466 controls [13]. Hu et al. leveraged 44 tissues, including ten brain tissues (anterior cingulate cortex BA24, caudate basal ganglia, cerebellar hemisphere, cerebellum, cortex, frontal cortex BA9, hippocampus, hypothalamus, nucleus accumbens basal ganglia, and putamen basal ganglia), to build gene expression prediction models using a new joint-tissue imputation approach under the proposed UTMOST framework, which aims to increase the prediction accuracy by borrowing information across tissues. By applying the models to 17,008 AD cases and 37,154 controls, they identified 12 novel susceptibility gene candidates [14]. In a recent TWAS by Gerring et al., gene expression prediction models of 48 tissues built using The Genotype-Tissue Expression (GTEx) project data (version 7) were developed, and 126 tissue-specific gene-based associations involving 50 genes were reported for AD risk [15]. These findings have contributed substantially to the etiological understanding of AD. However, some limitations of existing TWAS should be noted. First, most of these studies do not systematically evaluate different brain tissues [12, 13, 16]. It is known that multiple types of brain tissues could be causal for AD pathogenesis [5, 8, 15]. AD is a neurodegenerative disorder partly induced by dysregulation of different brain regions [17], which may affect the hypothalamus-pituitary-adrenal axis function leading to changes of behavior and mood in patients [18–20]. Although Hu et al [14] studied different brain tissues, they built gene expression prediction models with relatively small reference datasets (version 6 of GTEx), leading to a much smaller number of prediction models with satisfactory performance. Second, existing studies largely relied on earlier AD GWAS datasets with limited numbers of AD cases and controls for association analyses. Furthermore, although research supports an immune component in the etiology of AD [21, 22], existing TWAS have been limited in studying tissues, such as the spleen, having immune cell types. These limitations have constrained the ability of existing TWAS for characterizing AD-associated genes.

Herein, to identify novel candidate susceptibility genes for AD risk, we performed a comprehensive TWAS of AD risk using GWAS data involving 71,880 (proxy) cases and 383,378 (proxy) controls of European ancestry, by leveraging gene expression prediction models built using state-of-the-art modeling strategies in ten different tissues, from the latest version of The Genotype-Tissue Expression (GTEx) v8 [23], that are potentially related to AD pathogenesis [20, 24, 25]. It was identified earlier that AD-by-proxy, based on parental diagnoses, showed a high genetic correlation with AD (*r*_*g*_ = 0.81) [9]. Thus, we leveraged the meta-analysis results of the clinical AD GWAS and the AD-by-proxy GWAS in this study to increase the statistical power. The tissues analyzed here included brain cortex, anterior cingulate cortex BA24, hippocampus, amygdala, caudate basal ganglia, nucleus accumbens basal ganglia, putamen basal ganglia, substantia nigra, hypothalamus of cerebrum, and pituitary. Spleen tissue was also included in a separate analysis to characterize additional genes related to AD.

## Methods

### Building gene expression prediction models

Genome and gene expression data of ten different brain tissues and spleen tissue from the GTEx (v8) [23] were used to develop gene expression genetic prediction models. The detailed information of the GTEx v8 dataset including genotyping method, RNA sequencing experiments, and quality control processes, has been described elsewhere [26, 27]. In brief, only genes with a reasonable expression level were included for model building (thresholds: ≥0.1 TPM in ≥20% of samples and ≥6 reads (unnormalized) in ≥20% of samples). Expression values for each gene were inverse normal transformed across samples. By adjusting for the sex, platform, first five principal components, and PEER (Probabilistic Estimation of Expression Residuals) factors, the residual of normalized expression level was generated for model training. All 838 GTEx v8 samples (more than 85% are of European ancestry) were included. We included brain cortex (*n* = 205), anterior cingulate cortex BA24 (*n* = 147), hippocampus (*n* = 165), amygdala (*n* = 129), caudate basal ganglia (*n* = 194), nucleus accumbens basal ganglia (*n* = 202), putamen basal ganglia (*n* = 170), substantia nigra (*n* = 114), hypothalamus of cerebrum (*n* = 170), pituitary (*n* = 237), and spleen (*n* = 146) samples with matched genome and transcriptome data available for gene expression genetic model building using a modified UTMOST framework [14]. Single-nucleotide polymorphism (SNPs) located within 1 Mb upstream and downstream of the gene were included as potential features for the model building.

The weights for SNPs in the prediction model were estimated with a LASSO penalty both within- and cross-tissues. Fivefold cross-validation was performed for hyperparameter tuning using two hyperparameters, *λ*_1_ and *λ*_2,_ for the within-tissue and cross-tissue penalization, respectively. In the final step of the original UTMOST model building pipeline, a “heritable gene” was defined by the model’s prediction performance estimated in the entire dataset which was used to train the final model. Model training and performance evaluation in the same dataset may result in overestimation of the prediction performance [28]. The overestimation will result in a large number of low-quality “heritable genes” for downstream analysis, which will increase the false positive rate and the multiple comparison burden. To avoid the model estimation in the entire dataset (and thus avoid the inflated performance), we modified the model training process by using a consistent array of hyperparameter pairs across the five-fold cross-validation, which made the tuning error of hyperparameter pairs comparable across different folds in the cross-validation step (in contrast to the original UTMOST, which used a fold-specific array of lambda pairs). After the fivefold training, the lambda pair with the lowest average tuning error across the five folds was selected for final use. The performance of the prediction models was assessed by the correlation between the predicted and observed expression levels in the combined tuning set. The script for the modified version of UTMOST is available at https://github.com/gamazonlab/MR-JTI/blob/master/model_training/UTMOST/main_modified.r [29]. Only models with Pearson’s correlation *r* ≥ 0.1 and *P* < 0.05 were retained for the subsequent association analyses.

### Associations between genetically predicted gene expression levels and AD risk

Based on S-PrediXcan [10], we investigated the associations of genetically predicted gene expression in multiple tissues with AD risk by applying the prediction models to the summary statistics generated from a large GWAS of AD, which included 71,880 (proxy) cases and 383,378 (proxy) controls of European ancestry from three consortia (Alzheimer’s disease working group of the Psychiatric Genomics Consortium (PGC-ALZ), the International Genomics of Alzheimer’s Project (IGAP), and the Alzheimer’s disease Sequencing Project (ADSP)) and UK Biobank [9, 30]. The SNP-SNP covariance matrices estimated using all GTEx v8 subjects were used. For each gene, in the main analyses, we combined the association *p* values across the different brain tissues by a Cauchy distribution-based combination approach [31]. Briefly, we transformed the *P* values derived from TWAS of multiple tissues into standard Cauchy random variables and used the average of transformed *P* values as the test statistics. Its *P* value can be calculated analytically, which is highly accurate when the actual *P* value is very small. Cauchy combination test was conducted using R V3.6.1. software [32]. We then applied the Bonferroni correction to determine the significance threshold. Focusing on the identified associated genes, to determine the most likely causal genes for AD risk, we conducted FOCUS (Fine-mapping Of CaUsal gene Sets) fine-mapping analysis, as described elsewhere [33]. Briefly, we ran FOCUS in each type of brain tissue separately with GWAS summary statistics [9], TWAS results, and prediction models for each corresponding tissue as inputs. FOCUS outputted the posterior probability for each gene, and the default 90% credible gene set was used to determine the likely causal genes. We also conducted a separate analysis focusing on spleen tissue to identify additional genes showing an association with AD.

### “Core Analysis” in Ingenuity Pathway Analysis (IPA)

For the identified AD risk-associated genes using brain tissues in main analyses, we performed the “Core Analysis” in IPA [34] to assess the enriched pathways, biological function, or diseases and networks. Briefly, the list of identified AD risk-associated genes was submitted to IPA for “Core Analysis”.

## Results

### Brain tissue and spleen tissue gene expression prediction models

We developed gene expression prediction models using a modified UTMOST [14] (Unified Test for MOlecular SignaTures) method. The number of prediction models with a performance of at least 0.01 (i.e., the correlation between predicted expression and measured expression of at least 10%) ranged from 5015 to 8582 across the different brain tissues we assessed (Additional file 1: Table S1). There were 8759 models established with performance *R*^2^ ≥0.01 for the spleen tissue.

### Associations of predicted gene expression levels in brain tissues with AD risk

**Fig. 1:**
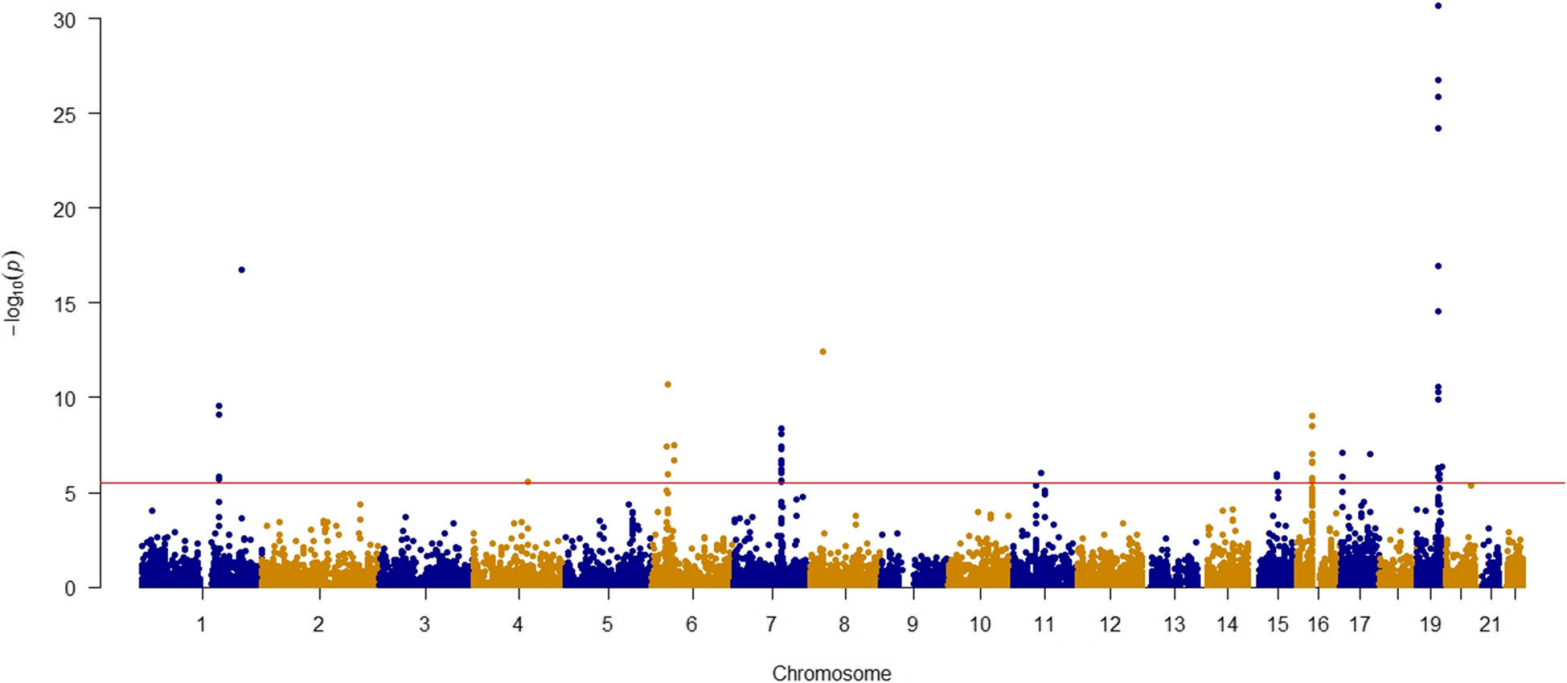
Manhattan plot of association results from the Alzheimer’s disease transcriptome-wide association study. The *x*-axis represents the genomic position of the corresponding gene, and the *y*-axis represents -log_10_-transformed association combined *P* value, which is derived from individual *p* values from single tissue model-based analyses. Each dot represents the association for one specific gene. The red line shows combined *P* = 3.38 × 10^−6^ based on 14,787 tests. The top two associations of *TOMM40* and *APOE* with *P* < 2.38 × 10^−134^ are not shown in this figure

The full results of TWAS for AD risk across the ten brain tissues were included in Additional file 2: Table S2. For each gene, we combined the gene-level association *p* values across the different brain tissues by a Cauchy distribution-based combination approach [31] and then used the stringent Bonferroni correction threshold to determine the significantly associated genes. Of the 14,787 genes tested, we observed 54 significant associations at Bonferroni corrected threshold *P* < 3.38 × 10^−6^ (Fig. 1). After excluding *HLA-DQA2* which is located in a linkage disequilibrium (LD)-extensive region, 53 genes located at 18 distinct genomic loci were retained (Table 1 and Additional file 3 and 4: Table S3 and S4).

**Table 1:** Thirty-five genes that have not been previously identified in TWAS for AD risk

Region	Gene name	Type^**a**^	Combined ***P*** value	Index SNP(s)^**b**^	Distance of gene to the index SNP (kb)
1q23.3	*NDUFS2*	Protein	2.81 × 10^−10^	rs4575098	11.50
	*FCER1G*	Protein	7.43 × 10^−10^	rs4575098	29.63
	*B4GALT3*	Protein	1.45 × 10^−6^	rs4575098	7.63
	*NIT1*	Protein	1.85 × 10^−6^	rs4575098	60.16
4q25	*FAM241A*	Protein	2.88 × 10^−6^	rs6448451	102,041.93
6p12.3	*AL355353.1*	lncRNA	3.16 × 10^−8^	rs9381563	12.34
	*CD2AP*	Protein	1.98 × 10^−7^	rs10948363	Within
6p21.32	*BTNL2*	Protein	1.95 × 10^−11^	rs9271192	203.63
6p21.33	*SAPCD1*	Protein	3.61 × 10^−8^	rs9271192	845.90
7q22.1	*PVRIG*	Protein	4.44 × 10^−9^	rs7384878	112.94
	*AZGP1*	Protein	3.96 × 10^−8^	rs7384878	358.38
	*AC004522.3*	lncRNA	5.00 × 10^−8^	rs7384878	385.81
	*CNPY4*	Protein	1.85 × 10^−7^	rs7384878	208.92
	*CASTOR3*	Protein	2.77 × 10^−7^	rs7384878	62.19
	*LAMTOR4*	Protein	3.35 × 10^−7^	rs7384878	178.48
	*STAG3*	Protein	5.62 × 10^−7^	rs7384878	112.94
	*GJC3*	Protein	8.58 × 10^−7^	rs7384878	404.81
	*GPC2*	Protein	2.31 × 10^−6^	rs7384878	157.05
	*AP4M1*	Protein	2.78 × 10^−6^	rs7384878	224.08
11q12.1	*FAM111A*	Protein	9.60 × 10^−7^	rs983392	1001.00
15q21.3	*AC090515.2*	lncRNA	1.13 × 10^−6^	rs442495	37.66
15q21.3-q22.1	*MINDY2*	Protein	1.43 × 10^−6^	rs442495	40.78
16p11.2	*KAT8*	Protein	9.70 × 10^−10^	rs59735493	Within
	*PRSS36*	Protein	2.96 × 10^−9^	rs59735493	17.15
	*AC135050.6*	lncRNA	9.24 × 10^−8^	rs59735493	Within
	*PRSS53*	Protein	2.25 × 10^−7^	rs59735493	32.15
	*ZNF668*	Protein	1.81 × 10^−6^	rs59735493	47.54
	*CCDC189*	Protein	2.16 × 10^−6^	rs59735493	359.56
	*AC135050.1*	lncRNA	2.66 × 10^−6^	rs59735493	58.98
17p13.2	*AC012146.1*	lncRNA	8.33 × 10^−8^	rs9916042	30.32
	*SCIMP*	Protein	1.47 × 10^−6^	rs9916042	127.77
17q23.3	*ACE*	Protein	8.67 × 10^−8^	rs28394864	14,103.65
19q13.31-13.32	*AC243964.3*	lncRNA	5.89 × 10^−25^	rs75627662	185.10
19q13.32	*GEMIN7*	Protein	5.79 × 10^−7^	rs75627662	168.88
	*OPA3*	Protein	1.12 × 10^−6^	rs76320948	136.37

^a^Protein: protein coding genes; lncRNA: long noncoding RNAs; ^b^The risk SNP closest to the gene is presented and a full list of all risk SNPs, and their distances to the genes are presented in Additional file 6: Table S6

These include 35 genes that have not been previously reported to be associated with AD risk in TWAS (Table 1 and Additional file 3: Table S3), as well as 18 genes previously reported in AD TWAS (Additional file 3: Table S3). The associations based on individual tissue prediction models can be found in Additional file 3 and 4: Table S3 and S4. A total of 45 genes showed concordant association directions across all the tested tissues, positively (17 genes) or negatively (28 genes). Tissue-specific association directions were observed for the remaining eight genes (*APOC2*, *APOC4*, *APOE*, *FAM111A*, *GPC2*, *LAMTOR4*, *OPA3*, and *ZNF112*). Based on the fine-mapping approach, FOCUS [33], using 90% credible gene sets to define putative causal genes, we found that 21 of the genes are likely causal genes for AD risk (Table 2 and Additional file 5: Table S5). Ten of the 21 putative causal genes (*NDUFS2*, *FCER1G*, *BTNL2*, *AC004522.3*, *GPC2*, *PVRIG*, *KAT8*, *AC012146.1*, *ACE*, and *AC243964.3*) have not been reported in previous TWAS.

**Table 2:** Fine-mapping results based on TWAS-identified genes for AD risk

Region		Tx Start	Tx End	Tissue	OR (95% CI)	TWAS *P* value	Focus^a^
1q23.3	*NDUFS2*	161166894	161184185	Brain caudate basal ganglia	0.96 (0.95–0.97)	1.83 × 10^−9^	0.96
				Brain anterior cingulate cortex BA24	0.95 (0.93–0.96)	6.79 × 10^−10^	1
				Brain hippocampus	0.94 (0.92–0.96)	3.29 × 10^−10^	1
	*FCER1G*	161185024	161190489	Brain hypothalamus	0.92 (0.89–0.94)	1.44 × 10^−9^	0.97
1q32.2	*CR1*	207669492	207813992	Brain hypothalamus	1.20 (1.15–1.25)	1.00 × 10^−16^	1
				Brain nucleus accumbens basal ganglia	1.17 (1.13–1.21)	1.44 × 10^−17^	1
				Brain anterior cingulate cortex BA24	1.13 (1.10–1.16)	2.47 × 10^−17^	1
				Brain caudate basal ganglia	1.13 (1.10–1.16)	1.22 × 10^−17^	1
				Brain hippocampus	1.12 (1.09–1.15)	6.76 × 10^−17^	1
				Brain amygdala	1.12 (1.09–1.15)	2.60 × 10^−17^	1
				Brain substantia nigra	1.12 (1.09–1.15)	2.28 × 10^−17^	1
				Brain cortex	1.11 (1.09–1.14)	7.96 × 10^−18^	1
				Brain putamen basal ganglia	1.11 (1.09–1.14)	1.41 × 10^−17^	1
6p21.32	*BTNL2*	32361740	32374905	Brain caudate basal ganglia	1.07 (1.05–1.10)	5.09 × 10^−12^	1
				Brain cortex	1.05 (1.03–1.07)	1.10 × 10^−6^	0.91
7q22.1	*AC004522.3*	99527015	99546243	Brain amygdala	0.96 (0.94–0.97)	2.63 × 10^−8^	0.93
	*GPC2*	99767229	99774995	Brain putamen basal ganglia	1.04 (1.02–1.05)	1.71 × 10^−6^	0.96
	*PVRIG*	99815864	99819113	Brain hippocampus	0.98 (0.97–0.99)	9.74 × 10^−9^	0.93
	*NYAP1*	100081550	100092422	Brain hypothalamus	0.86 (0.82–0.91)	2.72 × 10^−9^	1
8p21.1	*CLU*	27454434	27472548	Brain putamen basal ganglia	0.92 (0.90–0.94)	3.77 × 10^−13^	1
16p11.2	*KAT8*	31127075	31142714	Pituitary	0.98 (0.97–0.99)	1.28 × 10^−9^	0.98
				Brain hippocampus	0.98 (0.97–0.98)	4.90 × 10^−10^	0.96
				Brain caudate basal ganglia	0.98 (0.97–0.98)	1.43 × 10^−9^	0.93
				Brain amygdala	0.98 (0.97–0.98)	1.16 × 10^−9^	0.93
				Brain substantia nigra	0.97 (0.97–0.98)	1.64 × 10^−9^	1
				Brain anterior cingulate cortex BA24	0.97 (0.96–0.98)	3.59 × 10^−10^	0.98
17p13.2	*AC012146.1*	5014763	5017674	Brain caudate basal ganglia	0.97 (0.97–0.98)	5.29 × 10^−8^	0.96
				Brain hippocampus	0.97 (0.97–0.98)	1.98 × 10^−7^	0.96
				Brain amygdala	0.97 (0.96–0.98)	4.12 × 10^−8^	0.97
17q23.3	*ACE*	61554422	61599205	Brain anterior cingulate cortex BA24	0.97 (0.96–0.98)	5.18 × 10^−8^	0.99
19q13.31-13.32	*AC243964.3*	45135500	45222031	Brain amygdala	1.04 (1.03–1.05)	1.78 × 10^−25^	1
				Brain anterior cingulate cortex BA24	1.03 (1.03–1.04)	6.17 × 10^−25^	1
				Brain putamen basal ganglia	1.03 (1.02–1.03)	8.54 × 10^−25^	1
				Pituitary	1.03 (1.02–1.03)	1.17 × 10^−24^	1
19q13.31	*CEACAM19*	45165545	45187631	Brain amygdala	1.05 (1.04–1.06)	5.36 × 10^−20^	1
				Brain anterior cingulate cortex BA24	1.04 (1.03–1.05)	1.66 × 10^−15^	0.99
19q13.32	*NECTIN2*	45349432	45392485	Brain substantia nigra	0.92 (0.90–0.93)	2.67 × 10^−27^	1
				Pituitary	0.98 (0.96–0.99)	4.62 × 10^−3^	1
	*TOMM40*	45393826	45406946	Pituitary	9.05 (8.19–9.99)	0	1
	*APOE*	45409011	45412650	Brain substantia nigra	1.42 (1.38–1.46)	7.92 × 10^−135^	1
				Pituitary	0.86 (0.84–0.89)	4.89 × 10^−24^	1
	*APOC4*	45445495	45452820	Brain putamen basal ganglia	0.98 (0.98–0.99)	2.65 × 10^−12^	1
	*APOC2*	45449243	45452822	Brain anterior cingulate cortex BA24	1.08 (1.06–1.11)	7.84 × 10^−12^	1
				Brain putamen basal ganglia	1.03 (1.02–1.04)	2.77 × 10^−8^	1
				Pituitary	0.95 (0.93–0.97)	1.33 × 10^−5^	1
	*ZNF296*	45574758	45579846	Brain amygdala	0.89 (0.86–0.92)	2.90 × 10^−12^	1
	*KLC3*	45836692	45854778	Brain amygdala	0.94 (0.92–0.97)	1.49 × 10^−6^	0.94

^a^The posterior probabilities of FOCUS

The full list of GWAS identified risk SNPs for AD and their distances to the identified genes are shown in Additional file 6: Table S6. Of the 35 newly identified associated genes, four genes (*FAM241A* at 4q25, *SAPCD1* at 6p21.33, *FAM 111A* at 11q12.1, and *ACE* at 17q23.3) are more than 500 kb away from any GWAS-identified AD risk variants (Table 1 and Additional file 3: Table S3). Of the 18 previously reported AD-associated genes, the directions of the associations are consistent between the current study and previous TWAS studies (Additional file 4: Table S4).

In a separate analysis focusing on the spleen tissue, 26 significant associations at Bonferroni corrected threshold *P* < 5.71 × 10^−6^ (0.05/8759) were identified and 25 genes were retained after excluding *HLA-DQA2* (Additional file 7: Table S7). Nineteen of them, namely, *NDUFS2* (1q23.3), *FCER1G* (1q23.3), *NIT1* (1q23.3), *FAM241A* (4q25), *AL355353.1* (6p12.3), *CLU* (8p21.1), *AC090515.2* (15q22.1), *KAT8* (16p11.2), *PRSS36* (16p11.2), *VKORC1* (16p11.2), *ZNF668* (16p11.2), *PRSS53* (16p11.2), *AC135050.6* (16p11.2), *AC012146.1* (17p13.2), *AC243964.3* (19q13.31-13.32), *CEACAM19* (19q13.31), *PVR* (19q13.31), *APOC4* (19q13.32), and *TRAPPC6A* (19q13.32), were also identified in our main analyses using the brain tissue gene expression prediction models. Of the remaining six genes, three (*INPP5D* at 2q37.1, *MS4A2* at 11q12.1, and *MS4A4E* at 11q12.2) were suggested in previous GWAS for AD risk [7] and three genes (*SLC24A4* at 14q32.12, *CTSH* at 15q25.1, and *SETD1A* at 16p11.2) were reported to be associated with AD risk in previous studies [35–37].

### Pathway analysis

For the genes identified in the main analyses focusing on brain tissues, we performed the “Core Analysis” function within Ingenuity Pathway Analysis (Ingenuity System Inc, USA), including “Canonical Pathway,” “Disease and Functions,” and “Network” analyses. Fourteen of 53 associated genes (*ACE*, *APOC1*, *APOC2*, *APOC4*, *APOE*, *CD2AP*, *CLU*, *CR1*, *FCER1G*, *NECTIN2*, *PRSS36*, *PRSS53*, *PVR*, and *ZNF668*) were enriched in 11 canonical pathways (*P* < 0.05) (Additional file 8: Table S8**)**. These contain the neuroprotective role of THOP1 in Alzheimer’s disease (*P* = 1.70 × 10^−3^). Other canonical pathways are related to immune function, such as IL-12 signaling and production in macrophages (*P* = 2.75 × 10^−7^), LPS/IL-1 mediated inhibition of RXR function (*P* = 1.10 × 10^−3^) and natural killer cell signaling (*P* = 7.41 × 10^−3^).

**Fig. 2:**
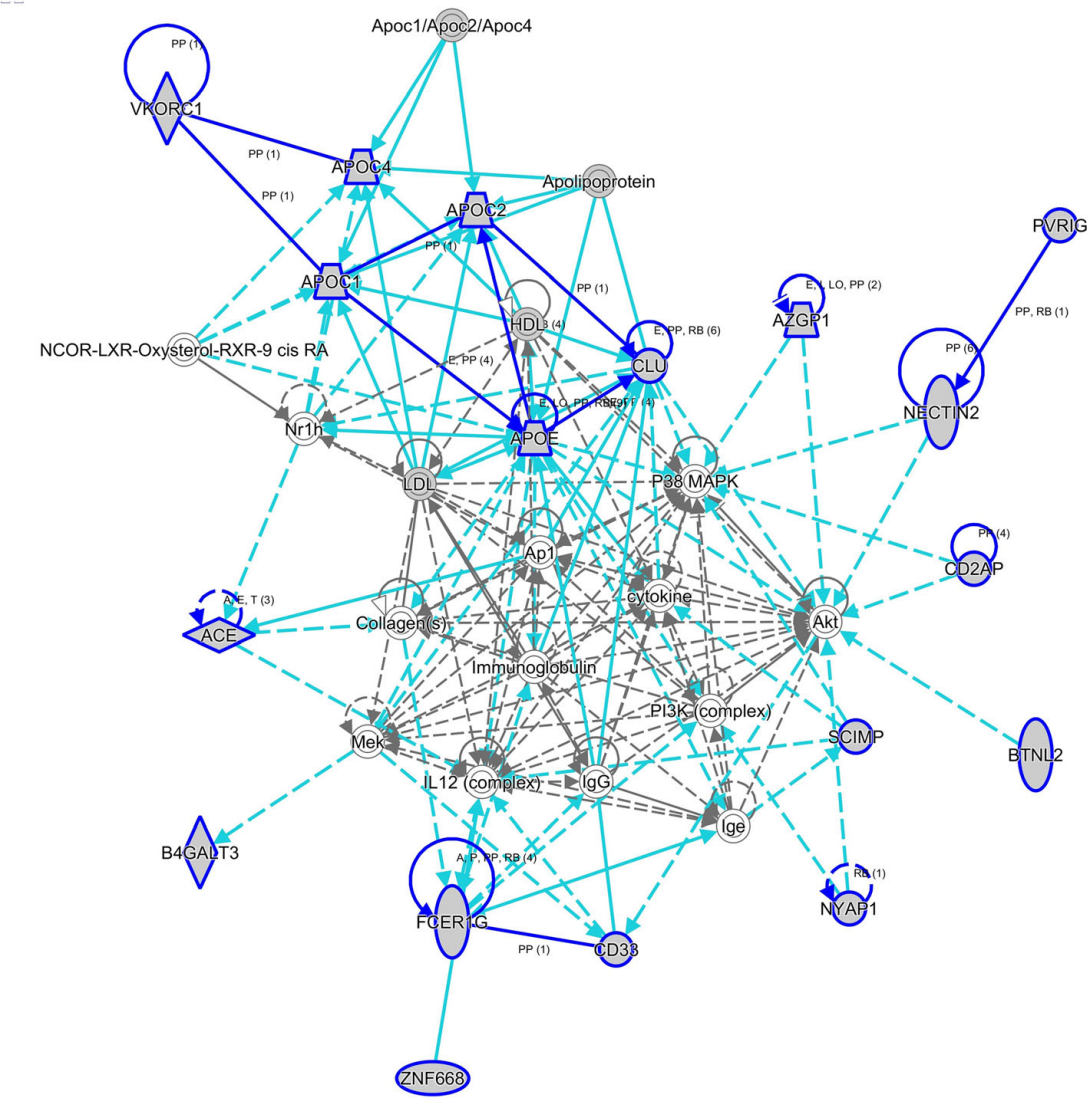
The top networks identified by Ingenuity Pathway Analysis (IPA). Function of the top network involved in metabolic disease, neurological disease, organismal injury and abnormalities. Circle indicates gene from the Knowledge Base—not part of our TWAS identified genes for AD risk. Shaded circle indicates our TWAS identified genes for AD risk. Straight line indicates direct interaction. Dashed line indicates indirect interaction. More information of IPA legend can be found in http://qiagen.force.com/KnowledgeBase/KnowledgeIPAPage?id=kA41i000000L5rTCAS

Overall, four networks were identified based on the Network Analysis (Additional file 9: Table S9). Eighteen associated genes were in the top network “Metabolic Disease, Neurological Disease, Organismal Injury and Abnormalities” (Fig. 2). Interestingly, some associated genes located in the network are known risk genes for AD, such as *CLU* (8p21.1) [38], *ACE* (17q23.3) [39], and *APOE* (19q13.32) [40], suggesting that the network could possibly regulate AD development.

Based on the “Disease and Functions” analysis, the top 20 disease functional categories can be found in Additional file 10: Table S10, including three categories related to AD, late-onset Alzheimer disease (*P* = 2.80 × 10^−11^), familial Alzheimer disease (*P* = 2.25 × 10^−6^), and Alzheimer disease (*P* = 4.16 × 10^−5^).

## Discussion

In this study, we built comprehensive gene expression prediction models leveraging a modified UTMOST method to systematically evaluate the associations of genetically predicated gene expression across the human transcriptome in ten brain tissues and spleen, a representative tissue that contains immune cell types, with AD risk. A total of 53 genes were found to be associated with AD risk for their genetically predicted expression in brain tissues, including 35 that have not been reported in previous TWAS. Fine-mapping analyses identified 21 of the 53 as putative causal genes for AD risk. Ten of the 21 fine-mapped genes are reported here for the first time. We also identified associations of specific genes in analyses of spleen tissue. Our findings contribute to improved understanding of the etiology and genetics of AD. Interestingly, different genes tend to be prioritized as putatively causal in different brain tissues. This may reflect that different causal genes may play a role in AD etiology in different brain tissues, which warrants further investigation.

Of the 35 AD-associated genes identified in analyses of brain tissues that have not been reported in previous TWAS, four of them, *FAM241A* at 4q25, *SAPCD1* at 6p21.33, *FAM 111A* at 11q12.1, and *ACE* at 17q23.3, are located at novel loci (Table 1). *ACE*, which encodes angiotensin I converting enzyme, is a known gene for AD [41, 42]. The remaining three genes, *FAM241A*, *SAPCD1*, and *FAM 111A*, are protein coding genes whose functions are not entirely clear and whose link with AD needs further investigation. Seven long noncoding RNA (lncRNA) genes (*AC004522.3*, *AC012146.1*, *AC090515.2*, *AC135050.1*, *AC135050.6*, *AC243964.3*, and *AL355353.1*) were also found to be associated with AD risk in this study. Previous work has suggested lncRNAs to potentially have a significant impact on normal neural development and on the development and progression of neurodegenerative diseases [43]. For example, specific lncRNAs may play a function as Decoy and/or Scaffold to sequester secretase enzyme, and thus decrease amyloid beta (Aβ) aggregation; they may also sequester kinases for decreasing tau hyperphosphorylation. Furthermore, lncRNAs may keep hyperphosphorylated tau proteins apart [44]. The lncRNAs identified here as associated with AD risk warrant further investigation.

The *APOE* has been identified as a biomarker for prognosis of mild cognitive impairment and AD [45] and for diagnosis of depressive disorder and dementia [46] and used as a biomarker for measuring the efficacy of testosterone in treating AD and mild cognitive impairment [47]. In our TWAS, interestingly, predicted expression of *APOE* in brain substantia nigra and caudate basal ganglia was positively associated with AD risk, and the predicted expression in the pituitary was inversely associated with AD risk. This implies that the expression levels of *APOE* in different brain regions may be related to different mechanisms of AD progression, which warrants further investigation. In previous TWAS, predicted expression of *APOE* in the skin was reported to be positively associated with AD risk [15]. *APOE* was also associated with AD risk by analyzing cross tissue models in a previous TWAS [13]. Similar to *APOE*, *APOC1* (19q13.32), identified in our study, has also been previously suggested as a potential biomarker for AD (Additional file 9: Table S9). The predicted expression of *APOC1* in the brain nucleus accumbens basal ganglia, pituitary, and adrenal gland was inversely associated with AD risk, consistent with the direction identified in previous TWAS [15].

Previous studies have suggested that AD is a neurodegenerative disease with an immune component [21, 22]. In order to illustrate whether or not genes in the spleen, a tissue containing immune cell types, may influence AD risk, we leveraged spleen tissue gene expression prediction models and identified twenty-five genes showing an association with AD risk. Most of them (19/25) were also identified in our main analyses using brain tissue gene expression prediction models. Interestingly, focusing only on the associated genes based on analyses of brain tissue prediction models, we observed enrichment of specific immune function-related canonical pathways, supporting potential roles of such immune-related genes in the etiology of AD.

In our study, we performed TWAS and TWAS fine-mapping by leveraging the summary statistics of a meta-analysis of AD GWAS and AD-by-proxy GWAS given the strong genetic correlation between AD and AD-by-proxy outcomes. To further evaluate the impact of this study design on our findings, we have performed TWAS separately using the results from GWAS of clinically diagnosed AD [39] and GWAS of AD-by-proxy outcome [48]. In these two separate analyses (Additional file 11: Table S11), as expected, directions of the associations were largely consistent compared with those of our main design, supporting the validity of our design.

There are several strengths of this study. Firstly, we used a modified UTMOST method to develop genetic prediction models for gene expression, which can increase power by jointly analyzing data from multiple genetically correlated tissues [14]. This is in contrast to single-tissue methods, including PrediXcan and TWAS/FUSION, which do not account for the similarity of genetic regulation across different tissues. In contrast to the original UTMOST framework, our modified framework used consistent hyperparameter pairs across the fivefold cross-validation in the model training process, which avoids the overestimation of model performance (Additional file 12: Figure S1). Secondly, in this study, we comprehensively assessed ten tissues (derived from the brain) with strong prior support for being related to AD pathogenesis, thus maximizing the possibility of identifying AD related genes. Thus, instead of using the ROSMAP/AMP-AD, PsychENCODE [13, 49], or the CommonMind Consortium [50, 51] resources, we leveraged GTEx data which provides a broad sampling of brain tissues. To our knowledge, our study is the most comprehensive TWAS of AD involving multiple disease-related tissues that have not been systematically evaluated before. Thirdly, in this study, we included 71,880 (proxy) AD cases and 383,378 (proxy) controls, which could provide high statistical power to detect associations. Previous work has supported that AD-by-proxy based on parental diagnoses showed a strong genetic correlation with AD (*r*_*g*_ = 0.81).

Several potential limitations also need to be acknowledged to interpret our findings. As with all other TWAS, we cannot exclude the possibility that some of the associations identified in this study may be false positives. Several potential reasons could explain this, such as correlated expression across individuals, correlated predicted expression, as well as shared regulatory variants [11]. On the other hand, we conducted fine-mapping analyses (using FOCUS) to identify the most likely causal genes. Additional experimental work would be needed to better characterize whether the identified genes may play a causal role in AD pathogenesis. Furthermore, further statistical confirmations and functional validations are needed for the genes showing inconsistent association directions across the tested tissues.

## Conclusions

In summary, in this large-scale study, we identified 21 putative causal genes, including 10 that have not been reported in previous TWAS, showing an association with AD risk for their predicted expression in brain tissues. Our study provides substantial new information to improve our understanding of the genetics and etiology of AD risk.

## Supplementary Information

**Additional file 1: Table S1.**. Internal performance of gene expression genetic prediction models of ten brain tissues.

**Additional file 2: Table S2.** Full results of TWAS for AD risk across ten brain tissues.

**Additional file 3: Table S3.** Individual tissue model derived associations for 35 genes newly identified in the present TWAS.

**Additional file 4: Table S4.** Eighteen expression-trait associations for genes identified in the present and previous TWAS.

**Additional file 5: Table S5.** FOCUS fine mapping analysis results of AD TWAS identified genes across ten train tissues.

**Additional file 6: Table S6.** Full list of risk SNPs and their distances with the associated genes.

**Additional file 7: Table S7.** Canonical pathway of identified genes by TWAS.

**Additional file 8: Table S8.** Networks of identified genes.

**Additional file 9: Table S9.** Top 20 disease functional categories of identified genes.

**Additional file 10: Table S10.** TWAS identified nine genes at Bonferroni correction level for AD risk using spleen tissue gene expression prediction model.

**Additional file 11: Table S11.** Comparison of results of main analyses (outcome of both clinically diagnosed AD and AD-by-proxy) with analyses of clinically diagnosed AD outcome and AD-by-proxy outcome.

**Additional file 12: Figure S1.** Prediction performance (*r*^*2*^) comparison between the training set (GTEx, brain fortal cortex BA9) and the test set (PsychENCODE, brain prefortal cortex).

**Additional file 13.** An R script for FOCUS fine-mapping analysis.

## Data Availability

Access to the complete results of the main analyses and the developed gene expression prediction models can be requested by submission of an inquiry to the senior authors. The datasets of GTEx are publicly available via dbGaP (www.ncbi.nlm.nih.gov/gap): dbGaP Study Accession: phs000424.v8.p2. The summary statistics of AD GWAS by Jansen et al. [9] can be downloaded under https://ctg.cncr.nl/software/summary_statistics. The scripts used in this study are available in Additional file 13 and at the following links: Cauchy combination test (https://github.com/yaowuliu/ACAT/blob/master/R/ACAT.R [52], and the modified version of the UTMOST (https://github.com/gamazonlab/MR-JTI/blob/master/model_training/UTMOST/main_modified.r [29].

## Abbreviations

AD: Alzheimer’s disease
DLPFC: Dorsolateral prefrontal cortex
FDR: False discovery rate
GTEx: Genotype-Tissue Expression
GWAS: Genome-wide association studies
IPA: Ingenuity Pathway Analysis
TWAS: transcriptome-wide association study
UTMOST: Unified Test for MOlecular SignaTures
