# Supplementary material for: A transcriptome-wide association study of Alzheimer’s disease using prediction models of relevant tissues identifies novel candidate susceptibility genes
Source: Genome Med. 2021 Sep 1;13:141. doi: 10.1186/s13073-021-00959-y (PMC8408990; doi:10.1186/s13073-021-00959-y)
Supplement: Supplementary file 12 — Additional file 12: Figure S1. Prediction performance (r2) comparison between the training set (GTEx, brain fortal cortex BA9) and the test set (PsychENCODE, brain prefortal cortex). [file 13073_2021_959_MOESM12_ESM.pdf]

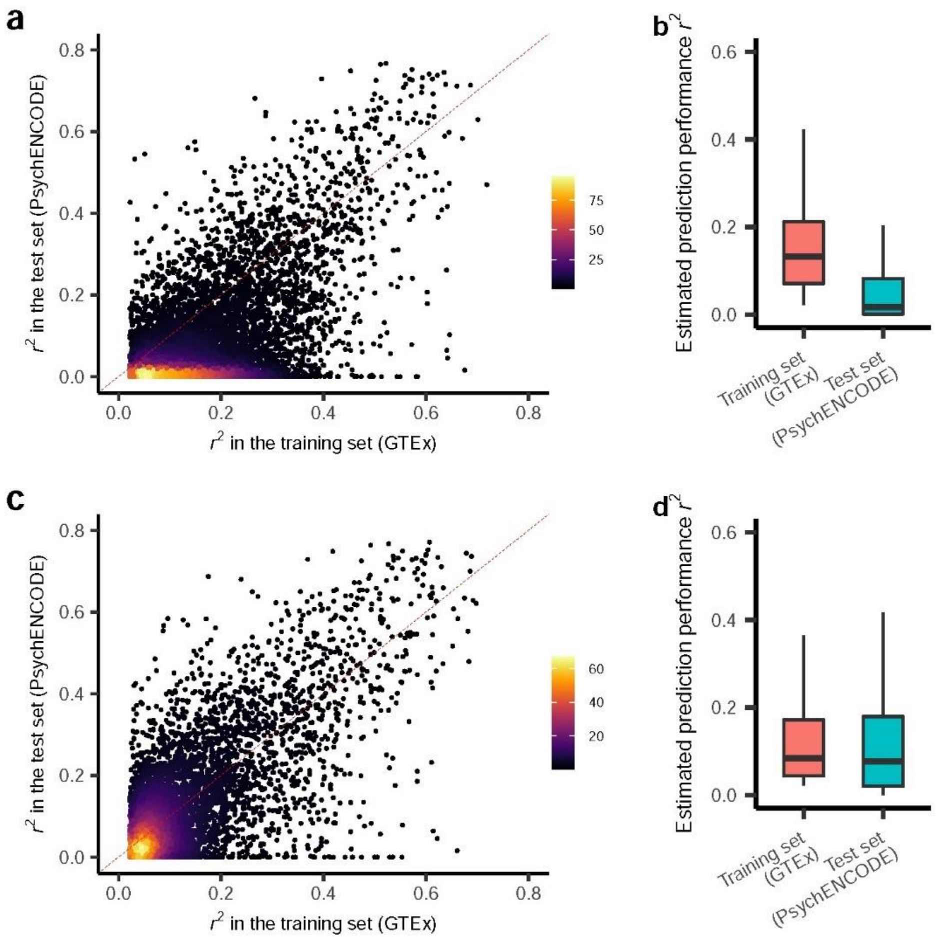

Figure S1. Prediction performance (r^2) comparison between the training set (GTEx, brain fortal cortex BA9) and the test set (PsychENCODE, brain prefortal cortex). The performance of the original UTMOST model (**a** and **b**) and that of the modified UTMOST model (**c** and **d**) were compared. The horizontal and vertical axes of the scatter plots (**a** and **c**) denote the prediction performance (r^2) estimated in GTEx (the training and cross-validation set) and in PsychENCODE (an external test data set), respectively. The yellow, red, and black dots indicate high, medium, and low density. The distribution of the prediction performance (r^2) is shown in the boxplot (**b** and **d**). The median of the performance is visualized as a black segment in the middle of the box. The lower and upper hinges correspond to the first and third quartiles (the 25th and 75th percentiles). The upper / lower whisker extends from the hinge to the largest / smallest value no further than / at most 1.5 × IQR from the hinge

(where IQR is the inter-quartile range or the distance between the first and third quartiles).
